# EGFR transcriptionally upregulates *UTX* via STAT3 in non-small cell lung cancer

**DOI:** 10.1007/s00432-021-03800-6

**Published:** 2021-10-18

**Authors:** Lin Zhou, Xiaomu Wang, Jingya Lu, Xiangning Fu, Yangkai Li

**Affiliations:** 1 Department of Thoracic Surgery, Tongji Hospital, Tongji Medical College, Huazhong University of Science and Technology, Wuhan, 430030 China; 2 Department of Clinical Pharmacy, Guangzhou First People’s Hospital, Guangzhou, 510180 China; 3 Department of Epidemiology and Biostatistics, School of Public Health, Tongji Medical College, Huazhong University of Science and Technology, Wuhan, 430030 China

**Keywords:** Histone demethylase UTX, Epidermal growth factor receptor, Tyrosine kinase inhibitor, Non-small cell lung cancer, Epigenetics

## Abstract

**Background:**

Histone demethylase UTX has been reported to participate in the occurrence and development of many cancers in tissue-specific manners. However, the role of UTX in non-small cell lung cancer (NSCLC) and exactly what regulates the expression of UTX remains unclear. Here, we analyzed the role of UTX in NSCLC in association with the widely recognized tumor driver epidermal growth factor receptor (EGFR).

**Methods:**

UTX levels in clinical samples were detected by immunohistochemistry staining, western blotting and real-time quantitative PCR. The expression of UTX in tumor tissue was correlated with the phosphorylation of EGFR. Cell proliferation and migration were evaluated by MTT and wound-healing assays. The impact of EGFR and its downstream pathways on UTX was explored with corresponding inhibitors, and examined by western blotting and real-time quantitative PCR.

**Results:**

In this study, we found that the expression of UTX in cancer tissues of patients with NSCLC was significantly higher than that in paracancerous tissues, and positively associated with EGFR phosphorylation levels. In addition, in NSCLC cell lines, UTX can promote proliferation and migration, while inhibition of its enzyme activity suppressed cell growth. Moreover, UTX expression was significantly upregulated when EGFR signaling pathway was activated, and vice versa when EGFR pathway was inhibited by tyrosine kinase inhibitor. Further mechanistic studies suggested that the activation of EGFR activated its downstream JAK/STAT3 signaling pathway and promoted STAT3 phosphorylation; the phosphorylated STAT3 transcriptionally promoted the levels of UTX.

**Conclusions:**

These results suggest an “EGFR-STAT3-UTX” axis that plays an oncogenic role in NSCLC.

**Supplementary Information:**

The online version contains supplementary material available at 10.1007/s00432-021-03800-6.

## Introduction

Lung cancer is the leading cause of cancer-related deaths globally (Sung et al. 2021). Due to the lack of obvious symptoms in the early stage of NSCLC, most patients have progressed to the middle and late stage when diagnosed, at which stage the survival rate is greatly reduced (Jin et al. 2020). Epidermal growth factor receptor (*EGFR*) mutation is one of the most classic and important carcinogenic mutations that induce NSCLC and drive its progression (da Cunha Santos et al. 2011). When EGFR binds to its specific ligand, it forms a dimer, which leads to autophosphorylation and activation of classical downstream signaling pathways of EGFR, including RAS/MAPK, PI3K/AKT and JAK/STAT3, thus promoting cell proliferation and avoiding apoptosis (Guo et al. 2015; Roskoski 2014). However, the carcinogenic mutation of *EGFR* will lead to the continuous activation of autophosphorylation, which can stimulate the abnormal proliferation of cells and lead to the occurrence and development of tumors (Liu et al. 2019). Tyrosine kinase inhibitor (TKI) can effectively block the kinase domain of EGFR and has a promising therapeutic effect on NSCLC with *EGFR* activating mutation. In recent years, the vigorous development of immunotherapy for lung cancer and new TKIs have significantly improved the prognosis of patients with advanced lung cancer, but the long-term survival is still poor due to the acquired drug resistance and relatively low response rate to TKIs and chemotherapeutic drugs (Herbst et al. 2018; Nagasaka and Gadgeel 2018; Zhang et al. 2019).

Histone methylation modification plays an important role in the occurrence and development of NSCLC and drug resistance in the treatment process (Chen et al. 2018). Ubiquitously transcribed X chromosome tetratricopeptide repeat protein (UTX; also known as lysine demethylase 6A, KDM6A) is a lysine demethylase. Its JumonjiC (JmjC) catalytic domain has histone demethylase activity, which specifically removes the dimethyl and trimethyl modification of lysine 27 of histone H3 (H3K27me2/3) (Gažová et al. 2019). In normal physiological processes, UTX plays an important role in growth and development, embryonic development and tissue-specific differentiation (Aumann and Abdel-Wahab 2014). Recent studies had shown that the role of UTX in cancer was tissue-specific (Schulz et al. 2019). UTX could promote the epithelial–mesenchymal transformation of breast cancer cells, enhanced the migration ability of breast cancer cells, and established metastatic foci of breast cancer (Taube et al. 2017). In addition, the expression of UTX was necessary for the survival of cervical cancer cells (Soto et al. 2017). Moreover, UTX, as a tumor suppressor, played an important role in various cancers such as lymphoma, human T cell acute lymphoblastic leukemia, pancreatic cancer and so on (Andricovich et al. 2018; Li et al. 2018; Ntziachristos et al 2014; Wang and Shilatifard 2019).

Since both UTX and EGFR signaling pathways play important roles in cancer, we aimed to clarify the role of UTX in NSCLC and the regulation mechanism between UTX and EGFR signaling pathway. Hence, we collected tissue samples from patients with NSCLC and detected the expression levels of UTX. And we studied the function and the upstream regulating mechanism of UTX via NSCLC cell lines.

## Methods

### Patients and tissue samples

Paired NSCLC and paracancerous tissue samples were collected from 40 patients who underwent pneumonectomy in the Department of Thoracic surgery, Affiliated Tongji Hospital of Huazhong University of Science and Technology Tongji Medical College. The clinicopathological data were collected and analyzed. Histological diagnosis of tumors was based on the WHO criteria (Travis et al. 2015). The study obtained the informed consent of the patient. The use of human tissue samples was approved by the Institutional Review Board of Tongji Hospital.

### Cell lines and cell culture

Human NSCLC cell lines H1975 (RRID:CVCL_1511), A427 (RRID:CVCL_1055), SK-LU-1 (RRID:CVCL_0629) and human embryonic lung fibroblast cell MRC5 (RRID:CVCL_0440) were obtained from the cell bank of Chinese Academy of Sciences. Human NSCLC cell lines A549 (RRID:CVCL_0023), H1299 (RRID:CVCL_0060) were obtained from Chinese typical culture preservation center (Wuhan, China). Human NSCLC cell lines H1975, A427, SK-LU-1 were cultured in RPMI-1640 medium containing 8% fetal bovine serum. Human NSCLC cell lines A549 and H1299 were cultured in DMEM high glucose medium containing 8% fetal bovine serum. All cell lines were cultured in 5% CO_2_ incubator at 37 ℃. Before being stimulated by cytokines such as EGF, the cells were starved for 16 h in corresponding culture medium containing 2% fetal bovine serum.

### Construction of plasmids and transfection

The expression plasmid with point mutation in *UTX* catalytic domain was constructed by substituting alanine for histidine at site 1146 and glutamate at site 1148. The RNAi plasmid was constructed based on pSUPER-retro-puro system, and the si*UTX* sequence for knockdown *UTX* is 5-GCATTTCAGGAGGTGCTTT-3. The plasmid was transfected into cells via Lipofectamine 2000 (Invitrogen, CA).

### Immunohistochemistry staining

The paraffin-embedded tissue sections were dewaxed and rehydrated, then heated by microwave in citrate buffer solution (90–95 ℃, 15 min) for thermal repair. After cooling, the slices were incubated with 3% H_2_O_2_ for 5 min to seal the endogenous peroxidase activity. Next, the slices were incubated with PBS for 5 min, and treated with 2% bovine serum albumin (diluted in PBST, i.e. PBS containing 0.1% Tween-20) for 15 min. Then, the primary antibody of UTX (1:500 dilution, Bethyl) was incubated overnight at 4 ℃. After washing three times with PBST for 5 min, the slices were incubated with the secondary antibody of UTX (1:500 dilution, Bethyl) at room temperature for 3 h. Next, VECTASTAIN ABC kit (Vector Laboratories, CA) and DAB kit (Vector Laboratories) were applied for staining. For each sample, three representative visual fields were photographed under microscope. UTX staining intensity was scored as follows: not detected (0), low (1), medium (2) and high (3). The percentage of UTX positive staining in the nucleus was scored as follows: 0-10% (0), 10-30% (1), 30-70% (2), and 70-100% (3). The immunohistochemical score of each visual field was acquired  by adding the score of UTX intensity with the score of UTX positive staining. The final immunohistochemical score of a sample was obtained by averaging the scores of its three visual fields. 

### MTT and wound-healing assays

The cells were placed into a 96-well plate at the density of 5000 cells per well and cultured for 24 h. After being treated with different doses of drugs for 24 h, it was replaced with 100 μL of 3-(4,5-dimethylthiazol-2-yl)-2,5-diphenyltetrazolium bromide (MTT; 0.5 mg/mL) per well, cultured in a 5% CO_2_ incubator at 37 ℃ for 4 h, and then 100 μL of formazen buffer was added to each well and incubated in 37 ℃ incubator for 6 h. Finally, the absorbance under 570 nm was measured by enzyme labeling instrument. In wound-healing assays, same number of cells in each group were placed into a 12-well plate, and on the next day, cells were evenly scratched to make a wound, and PBS was used to remove the scratched cells, and then replaced with 500 μL serum-free medium to eliminate the effect of cell proliferation. Then, under the microscope, images were taken and recorded at 0 h, 12 h and 24 h. Finally, Image Pro Plus is used to quantify the scratched area in the image.

### Western blotting

The cells were lysed with RIPA protein lysate (Beyotime, Shanghai, China), and the samples with appropriate protein concentration were heated at 95 ℃ for 10 min. Proteins were separated by sodium dodecyl sulfate–polyacrylamide gel electrophoresis (SDS-PAGE) and transferred to PVDF membrane (Millipore, MA). After sealing with 5% skim milk at room temperature for 30 min, the PVDF membranes were incubated with the primary antibody at 4℃ overnight. The next day, the membranes were washed with TBST for three times and incubated with the corresponding secondary antibody for 3 h. After washing the membranes for three times, signals were examined with chemiluminescence substrate (Advansta, CA). Information of antibodies is provided in Table S1.

### RNA extraction and real-time quantitative PCR

RNA ISO TM plus (Takara, Japan) was applied to extract total RNA from tissue samples or NSCLC cell lines. Then, M-MLV enzyme and other reverse transcription-related reagents (Takara, Japan) were used to reverse transcribe the total RNA into cDNA. Then, SYBR Green Master Mix was used to carry out quantitative PCR reaction in Bio-Rad CFX96 TouchTM real-time PCR detection system instrument. *ACTIN* cDNA was used as an internal reference. The primer sequences of qPCR used in this study are listed as follows:

*ACTIN* forward primer 5'-GGCTACAGCTTCACCACCAC-3' and reverse primer 5'-TAGCTCTTCTCCAGGGAGGA-3'.

*UTX* forward primer 5'-GCTGGAACAGCTGGAAAGTC-3' and reverse primer 5'-GAGTCAACTGTTGGCCCATT-3'.

### Immunofluorescence staining

The cells were incubated with the primary antibodies of UTX or p-STAT3 in PBS containing 1% BSA (1: 250) at 4 °C overnight and then incubated with the corresponding secondary antibody (1: 200) for 1 h the next day. DAPI (Invitrogen) was used to visualize the nucleus. Pictures were taken through a upright fluorescence microscope (Olympus, Japan).

### Statistical analysis

All the results were expressed as mean ± standard error (average ± SEM). Quantity One software was used for grayscale quantitative analysis of Western blotting results. Image Pro Plus software was used for quantitative analysis of microscope photographs. Student’s t test was used to analyze the significance of differences between groups. The results were considered to be statistically significant when *P* < 0.05.

## Results

### UTX is associated with EGFR signaling pathway in NSCLC

**Fig. 1:**
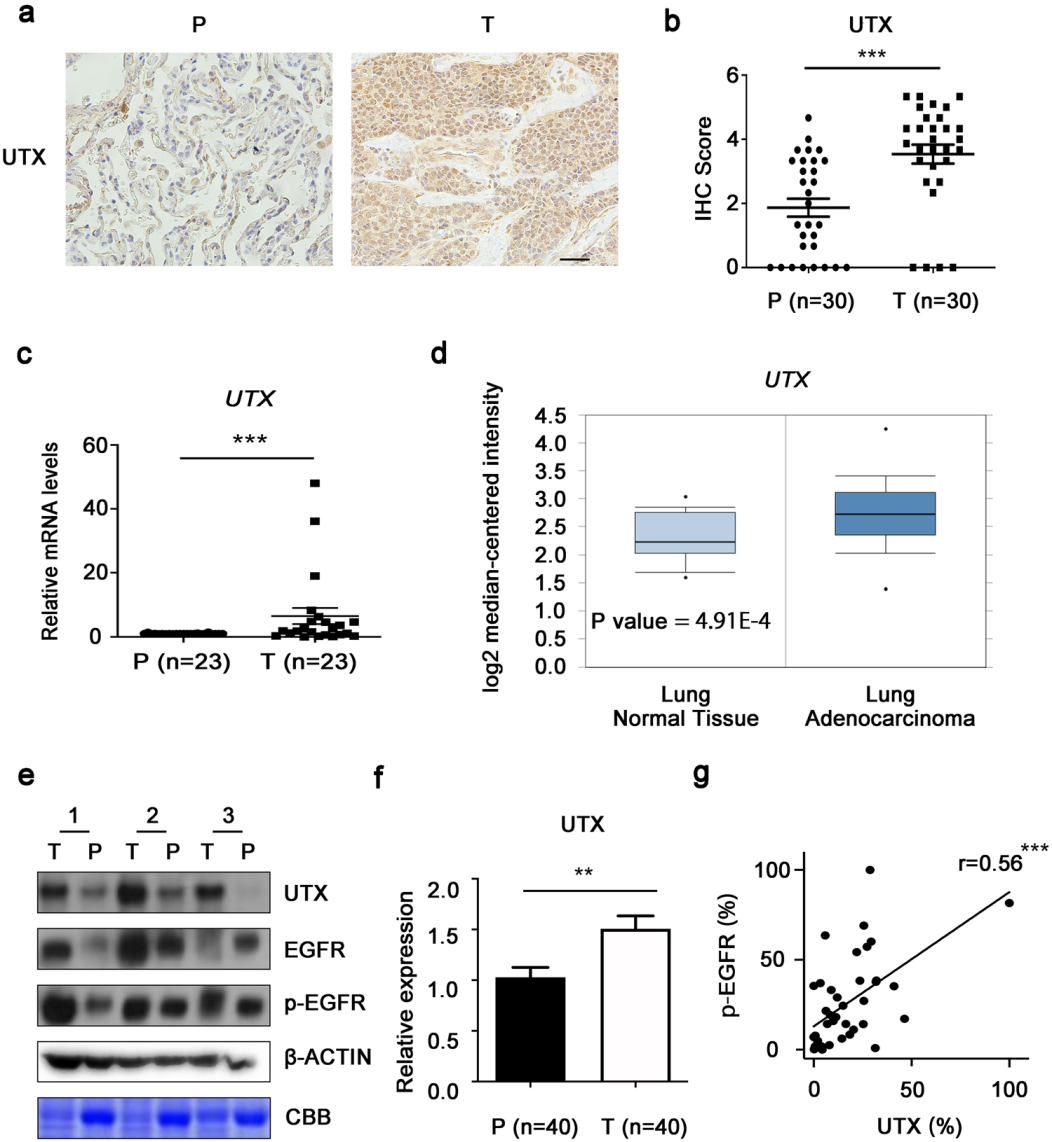
UTX is positively associated with EGFR phosphorylation in NSCLC tissues. **a** Representative immunohistochemistry (IHC) results of UTX in para-tumor and tumor tissues. **b** IHC score results show that UTX is upregulated in tumor tissues. **c** Transcriptional levels of *UTX* in para-tumor and tumor tissues. **d** The expression of *UTX* in NSCLC tumors and lung normal tissues (GSE31210) as analyzed by Oncomine. **e** Immunoblotting results of UTX in paired para-tumor and tumor tissues from three random NSCLC patients. **f** Quantification analysis of immunoblotting results of UTX in para-tumor and tumor samples. **g** Pearson correlation coefficient analysis shows that UTX protein levels and EGFR phosphorylation levels are positively associated in tumor samples. *P* para-tumor; *T* tumor; *CBB* Coomassie brilliant blue. Scale bar, 100 μm. Data, means ± SEM, *P < 0.05, **P < 0.01, ***P < 0.001, *NS* no significance

In order to investigate the relationship between the expression of UTX and EGFR signaling pathway in NSCLC, we first detected the expression levels of UTX in clinical samples of NSCLC. We detected the nuclear UTX signals in tumor and corresponding paracancerous tissues from 30 random NSCLC patients by immunohistochemistry (Fig. 1a and Supplementary Table S2). The expression levels of UTX in NSCLC tissues were significantly higher than that in corresponding paracancerous tissues (Fig. 1b). In addition, the mRNA levels of *UTX* in 23 random paired NSCLC and paracancerous tissues were detected by qPCR, and the mRNA expression levels of *UTX* were relatively high in NSCLC tissues (Fig. 1c). Consistently, analysis of a set of public data (GSE31210) (Okayama et al. 2012) via Oncomine (www.oncomine.org) also suggested that the level of *UTX* in tumor tissues is significantly higher than that of lung normal tissues (Fig. 1d). At the same time, further western blotting showed that the protein expression levels of UTX in 40 NSCLC tissues was significantly higher than that in the corresponding paracancerous tissues (Fig. 1e, f and Supplementary Table S3). Then, the protein levels of UTX and the levels of EGFR phosphorylation in the 40 NSCLC samples were analyzed by Pearson correlation coefficient after quantification, and a positive correlation between UTX and p-EGFR was found (Fig. 1g). These results suggest that UTX may play a tumorigenic role in NSCLC. And there is some unrevealed relationship between UTX and EGFR signaling pathway.

### UTX promotes the proliferation and migration of NSCLC cells

**Fig. 2:**
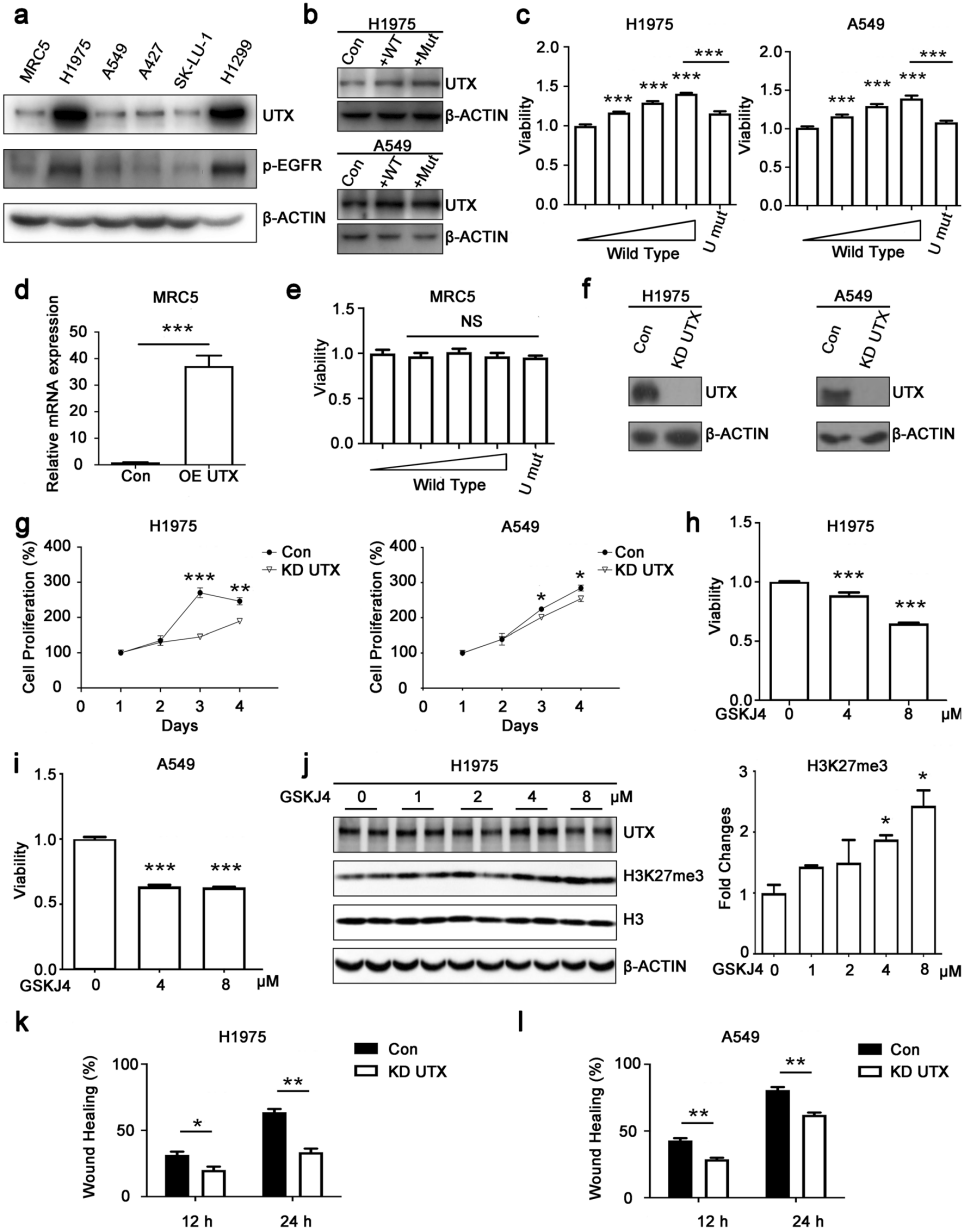
UTX promotes proliferation and migration of NSCLC cells. **a** UTX protein levels in human embryonic lung fibroblast MRC5 and five human NSCLC cells. **b** Overexpression of wild type UTX (WT) and mutant UTX (Mut) in NSCLC cells. **c** 48 h after transfection, wild-type UTX promotes cell proliferation in a dose-dependent manner (0, 25, 50 and 100 ng per well), whereas same amount of mutant UTX (U mut, 100 ng per well) shows no such effect. **d**, **e** Overexpression of wild-type or mutant UTX shows no effect on non-tumor cell proliferation. **f**, **g** Knockdown of UTX slows down cell proliferation rate. **h**–**j** 24-h treatment of 4 μM or 8 μM GSKJ4 shows cytotoxicity to NSCLC cells and upregulates H3K27me3 levels. **k**, **l** Wound healing assay shows knockdown of UTX inhibits migration of NSCLC cells. *Con* control group; *KD* knockdown; Data, means ± SEM. *P < 0.05, **P < 0.01, ***P < 0.001, *NS* no significance, comparing to control group unless specifically indicated

We performed western blotting to detect UTX expression in lung fibroblast cell MRC5 and different NSCLC cell lines (Fig. 2a). Finally, H1975 cell line with high expression of UTX and A549 cell line with low expression of UTX were selected for further study. First, in order to explore the effect of UTX on proliferation and whether the effect is associated with the JmjC catalytic domains of UTX, we constructed plasmids overexpressing wild-type *UTX* and mutant *UTX* without histone demethylase activity and transfected them into H1975 and A549 cells (Fig. 2b). The results showed that UTX could promote the proliferation of NSCLC cells in a dose-dependent manner, and such effect was dependent on the JmjC catalytic domain (Fig. 2c). In contrast, the proliferation of normal human lung cell MRC5 was not affected by these plasmids (Fig. 2d, e). Meanwhile, we further constructed a UTX knockdown plasmid, established UTX knockdown H1975 cell line and UTX knockdown A549 cell line and detected the effect of knockdown UTX on the proliferation of NSCLC cells (Fig. 2f). As expected, after knocking down UTX, the proliferation rate of NSCLC cells was significantly inhibited (Fig. 2g).

We also used GSKJ4 (a small molecular inhibitor that inhibits the activity of H3K27 demethylase) to treat NSCLC cells with gradient concentration to verify that UTX promotes the proliferation of NSCLC cells through its demethylase activity (Kruidenier et al. 2012). We found that the cell proliferation was significantly inhibited in the concentration range of 4–8 μM, and the levels of H3K27me3 increased in this concentration range, which coincided with our assumption (Fig. 2h–j). These results suggested that UTX promotes the proliferation of NSCLC cells through its JmjC domain.

Next, we explored the effect of UTX on the migration of NSCLC cells. We performed wound healing assays with H1975 UTX knockdown cell lines and A549 UTX knockdown cell lines and found that UTX knockdown could suppress the migration ability of H1975 and A549 cells (Supplementary Fig. S1a, b and Fig. 2k, l). To sum up, these experimental results show that UTX can promote the proliferation and migration of NSCLC cells and has tumor-promoting activity.

### JAK/STAT3 signaling pathway regulates UTX transcription in NSCLC cells

**Fig. 3:**
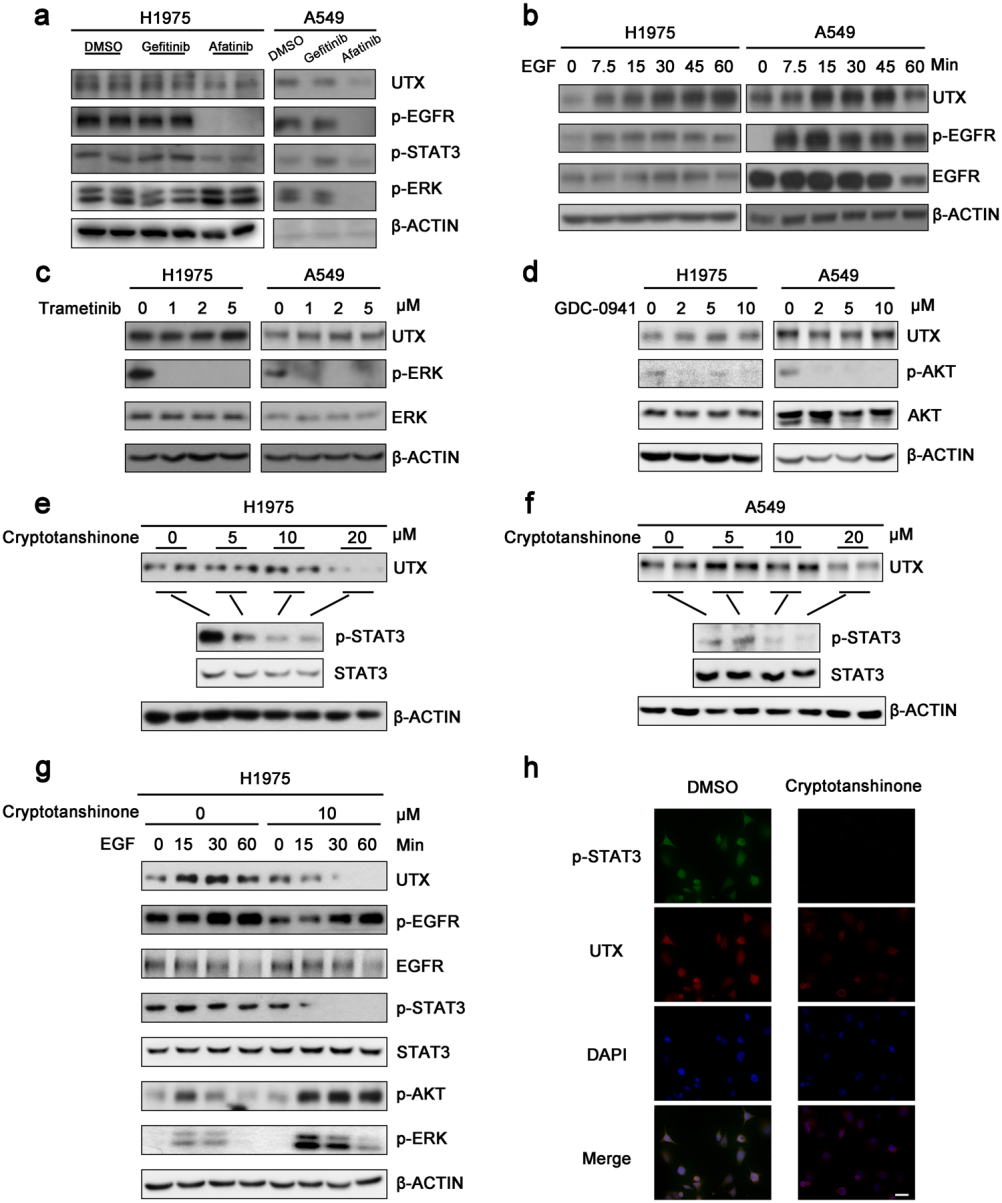
EGFR and STAT3 inhibition down regulate UTX. **a** EGFR pathway and UTX levels upon 48-h treatment of 0.1 μM Gefitinib or Afatinib. **b** EGF stimulation (100 ng/mL) leads to rapid increase of UTX. **c**, **d** Inhibiting MAPK pathway using 2-h Trametinib treatment or inhibiting PI3K/AKT pathway using 2-h GDC-0941 treatment show no significant effect on UTX levels. **e**, **f** 2-h Cryptotanshinone treatment down regulates UTX. **g** 2-h pre-treatment of 10 μM Cryptotanshinone inhibited EGF-induced increase of UTX. EGF concentration, 100 ng/mL. **h** Immunofluorescence assay shows decreased signals of STAT3 phosphorylation (green) and UTX (red) upon Cryptotanshinone treatment. Scale bar, 100 μm

A positive correlation between phosphorylated EGFR and UTX was found in previous clinical samples. However, we did not know whether there was a regulation between EGFR signaling pathway and UTX. In order to further explore the mechanism between UTX expression and EGFR signaling pathway in NSCLC, we used Gefitinib and Afatinib to treat H1975 cells and A549 cells (Li et al. 2008; Wakeling et al. 2002). Lung cancer cells harboring wild-type EGFR (A549) and T790M mutant EGFR (H1975) are more sensitive to Afatinib than to Gefitinib (Ninomiya et al. 2013). At the concentration of 0.1 μM, Afatinib, but not Gefitinib, significantly inhibited EGFR phosphorylation in H1975 and A549 cells, providing a case to investigate UTX levels under either ineffective or effective TKI treatment. As shown in Fig. 3a, when the EGFR signaling pathway was inhibited, the protein levels of UTX was significantly down-regulated along with the down-regulation of phosphorylated EGFR. We further stimulated H1975 cells and A549 cells with EGF, which is the ligand of EGFR. As expected, with the activation of EGFR signaling pathway, the level of phosphorylated EGFR was up-regulated, and the level of UTX was also significantly up-regulated (Fig. 3b). According to these results, we infer that EGFR could promote UTX, possibly through a downstream signaling pathway.

We then used Trametinib to inhibit MAPK signaling pathway, and GDC-0941 to inhibit PI3K/AKT signaling pathway (Haagensen et al. 2012; Ruscetti et al. 2018), and observed no changes in UTX levels (Fig. 3c, d), whereas after the inhibition of JAK/STAT3 signaling pathway, the expression of UTX decreased significantly (Fig. 3e, f). Treatment of Cryptotanshinone started at the dose of 5 μM since it inhibits JAK/STAT3 pathway with an IC_50_ value of 4.6 μM (Shin et al. 2009) and significantly inhibits STAT3 phosphorylation in both cell lines at the dose of 10 μM (Fig. 3e, f). Therefore, in further investigation to verify the effect of STAT3 on UTX, we treated H1975 cells with EGF after pre-treatment of 10 μM Cryptotanshinone. The results showed that the up-regulation of UTX by EGF could be blocked by Cryptotanshinone (Fig. 3g). At the same time, immunofluorescence assay also showed that the decrease of STAT3 phosphorylation was combined with the down-regulation of UTX (Fig. 3h).

**Fig. 4:**
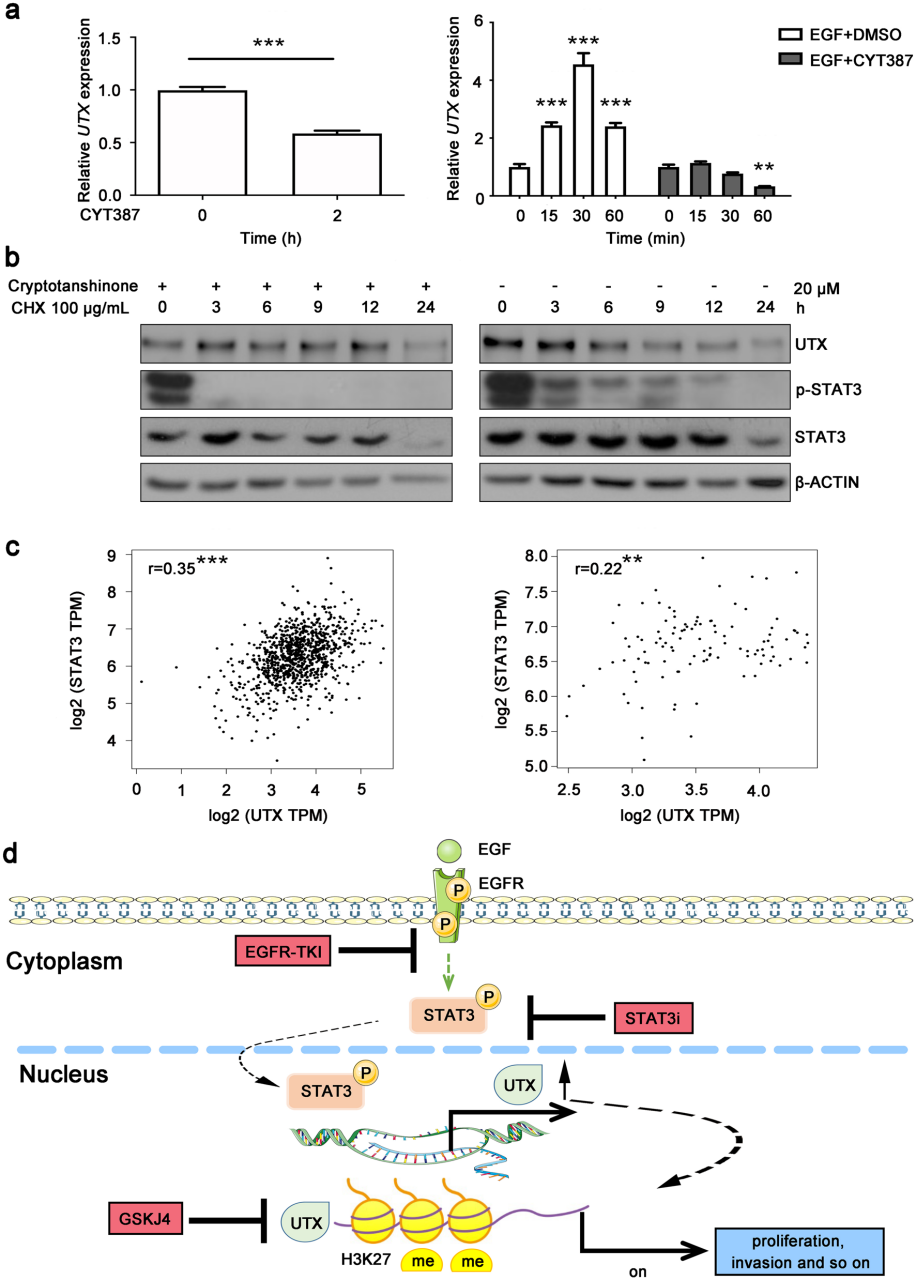
STAT3 transcriptionally regulates UTX and shows positive correlation with UTX in NSCLC samples. **a** 2-h treatment of JAK/STAT3 inhibitor CYT387 down regulates *UTX* mRNA levels (left panel) and inhibits EGF-induced transcription of *UTX* (right panel). Each group was compared to corresponding group collected at 0 h. **b** 2-h pre-treatment of Cryptotanshinone and CHX shows no effect on UTX protein stability. **c** Pearson correlation coefficient analysis on TCGA database by GEPIA (http://gepia.cancer-pku.cn/) shows that UTX positively associates with STAT3 in either tumor sample (left panel) and normal tissue samples (right panel). TPM, transcripts per million. **d** A working model of EGFR-STAT3-UTX axis in NSCLC. EGFR pathway activates its downstream transcriptional regulator STAT3 to up-regulate *UTX* mRNA and protein levels; UTX upregulation promotes proliferation and migration of NSCLC. Inhibiting UTX demethylation enzyme activity by GSKJ4 can suppress the proliferation of NSCLC cells. Data, means ± SEM. *P < 0.05, **P < 0.01, ***P < 0.001

Nevertheless, the exact mechanism of how STAT3 regulates the expression of UTX is unknown. STAT3 is well known as a transcription factor that defines gene expression programs in cancer (Yu et al. 2014). When it is phosphorylated and activated, it moves into the nucleus to promote target gene transcription (Yu et al. 2014). In order t to confirm whether *UTX* is a target gene of STAT3, we analyzed the promoter sequence of human *UTX* through JASPAR (http://jaspar.genereg.net/) and found that there were 22 sequences in the promoter of *UTX* that could bind to STAT3, and 5 top-scored sites were provided in Fig. S2. At the same time, we treated H1975 with CYT387, which was a blocker of JAK/STAT3 signaling pathway (Monaghan et al. 2011) and EGF. Through qPCR, we found that the *UTX* mRNA levels were increased after EGF stimulation. However, this phenomenon did not occur when JAK/STAT3 signaling pathway was blocked, and the *UTX* mRNA levels was even decreased (Fig. 4a). Then the cells were treated with protein synthesis inhibitor CHX and Cryptotanshinone (Park et al. 2019).  The results demonstrated that regardless of whether STAT3 phosphorylation was inhibited or not, the degradation of UTX was not affected (Fig. 4b). These results show that STAT3 regulates UTX on transcriptional levels, but not via affecting protein stability. Finally, we analyzed the Pearson correlation of *STAT3* and *UTX* using data of NSCLC and normal lung samples from The Cancer Genome Atlas database and found that there was a stronger correlation between *STAT3* and *UTX* levels in NSCLC tissues (Fig. 4c). In general, these results demonstrate that JAK/STAT3 signaling pathway regulates *UTX* transcription in NSCLC.

## Discussion

Ultimately, we found that in NSCLC cells, the JAK/STAT3 signaling pathway can promote *UTX* transcription, thus promoting the proliferation and migration. Accumulating studies indicated UTX plays a key role in the occurrence and development of many cancers (Barrows et al. 2020; Gozdecka et al. 2018; Schulz et al. 2019), but the functional mechanism of UTX in NSCLC remains unclear, and the understanding of the network of upstream regulatory mechanisms of UTX is still unknown. Therefore, our study mainly focuses on exploring the relationship between UTX and EGFR pathway. Our results indicated that EGFR c*a*n regulate the expression of UTX in NSCLC. Specifically, we found that when EGFR binds to its ligands, the intracellular domain was phosphorylated, which activated tyrosine kinase activity and JAK/STAT3 pathway and induced phosphorylation of STAT3. Activated STAT3 moved into the nucleus and induced *UTX* transcription, up-regulating the intracellular levels of UTX (Fig. 4d). Further verification on which promoter sequence STAT3 binds to *UTX* and how phosphorylated STAT3 r*e*gulates *UTX* transcription is needed.

UTX performs different functions in different types of cancer including lung cancer (Schulz et al. 2019; Wang and Shilatifard 2019). Wu et al. reported that UTX, as a tumor suppressor, was an important epigenetic regulator in lung tumorigenesis (Wu et al. 2018). However, Leng et al. reported that UTX and KMT2B jointly regulated the transcriptional procedures of related genes in NSCLC to promote carcinogenic phenotype (Leng et al. 2020). And our study found that UTX could promote the proliferation and migration of NSCLC cells through its H3K27 demethylase activity. Interestingly, even though our results with Leng showed that UTX played an oncogenic role in NSCLC, the mechanism by which UTX promotes cancer was inconsistent. Different results from previous studies together suggest that the roles of UTX in cancer are tissue-specific and diverse. For examples, UTX loss is a driver of bladder cancer (Nickerson et al. 2014); UTX cooperates with MLL4 to promote cell proliferation and invasiveness in breast cancer (Kim et al. 2014); while another study reports that UTX inhibits breast cancer stem cell properties, suggesting its tumor surpressing role (Choi et al. 2015). Similarly, UTX played a different role in NSCLC, acting as a tumor suppressor in some parts of NSCLC and as an oncogene in other parts (Leng et al. 2020; Wu et al. 2018). As for how to classify the subtypes of NSCLC according to the function of UTX, it is worthwhile for us to continue our exploration.

TKIs are new breakthroughs developed in recent decades for NSCLC treatment (Zarogoulidis et al. 2016). However, due to acquired drug resistance caused by various mechanisms, including histone modification, the efficacy of TKIs would been greatly reduced after 1-year medication (Camidge et al. 2014; Lehmann et al. 2019; Lovly and Shaw 2014). Moreover, the effect of TKIs combined with chemotherapy is not satisfactory (Rossi et al. 2017). Our results showed that UTX was a molecule of the downstream network of EGFR, and GSKJ4 showed anti-cancer effect. Thus, we speculated that UTX, which is considered as an oncogene in NSCLC, is expected to be a target for the treatment of some NSCLC cases that lack specific targeting treatment, and the combination of UTX inhibitor and TKIs may have positive effect on *EGFR* mutant NSCLC.

In brief, we proved that UTX played an oncogenic role in NSCLC. Moreover, STAT3 downstream of EGFR signaling pathway could promote the transcription of UTX, suggesting an “EGFR-STAT3-UTX” axis that participates in the progression of NSCLC, and can at least partly explain the higher levels of UTX in NSCLC tumor tissues comparing to normal samples. These results suggest that UTX is expected to be a target for the treatment of NSCLC, and more verification studies need to be performed.

## Supplementary Information

Below is the link to the electronic supplementary material.

Supplementary file1 (DOCX 404 kb)

## Data Availability

Data are available from the corresponding author on reasonable request.
