# Supplementary material for: EGFR transcriptionally upregulates UTX via STAT3 in non-small cell lung cancer
Source: J Cancer Res Clin Oncol. 2021 Oct 18;148(2):309–19. doi: 10.1007/s00432-021-03800-6 (PMC8800904; doi:10.1007/s00432-021-03800-6)
Supplement: Supplementary file 1 — Supplementary file1 (DOCX 404 kb) [file 432_2021_3800_MOESM1_ESM.docx]

*JOURNAL OF CANCER RESEARCH AND CLINICAL ONCOLOGY*

**Supplementary Materials**

*for*

**EGFR transcriptionally upregulates UTX via STAT3 in non-small cell lung cancer**

Lin Zhou^1#^, Xiaomu Wang^2#^, Jingya Lu^3^, Xiangning Fu^1^, Yangkai Li^1*^

^1^Tongji Hospital, Tongji Medical College, Huazhong University of Science and Technology, Wuhan, China, 430030

^2^Department of Clinical Pharmacy, Guangzhou First People's Hospital, Guangzhou, China, 510180

^3^Department of Epidemiology and Biostatistics, School of Public Health, Tongji Medical College，Huazhong University of Science and Technology, Wuhan, China, 430030

^#^ These authors contributed equally to this work.

*^*^ Corresponding author*

Yangkai Li, MD, Ph.D.

Tongji Hospital

Huazhong University of Science and Technology

Wuhan, China, 430030

E-mail: [doclyk@163.com](mailto:doclyk@163.com)

**The supplementary materials include 2 supplementary figures and 3 supplementary tables.**

**Supplementary figures**

Figure S1. Representative results of wound healing assay.

Figure S2. Five highest-rated predicted STAT3-binding sites on UTX promoter, as analyzed by JASPAR (<http://jaspar.genereg.net/>).

**Supplementary tables**

Table S1. List of antibodies in the present study.

Table S2. Features and IHC score of paired NSCLC and paracancerous samples.

Table S3. Features of 40 paired NSCLC and paracancerous samples.

**
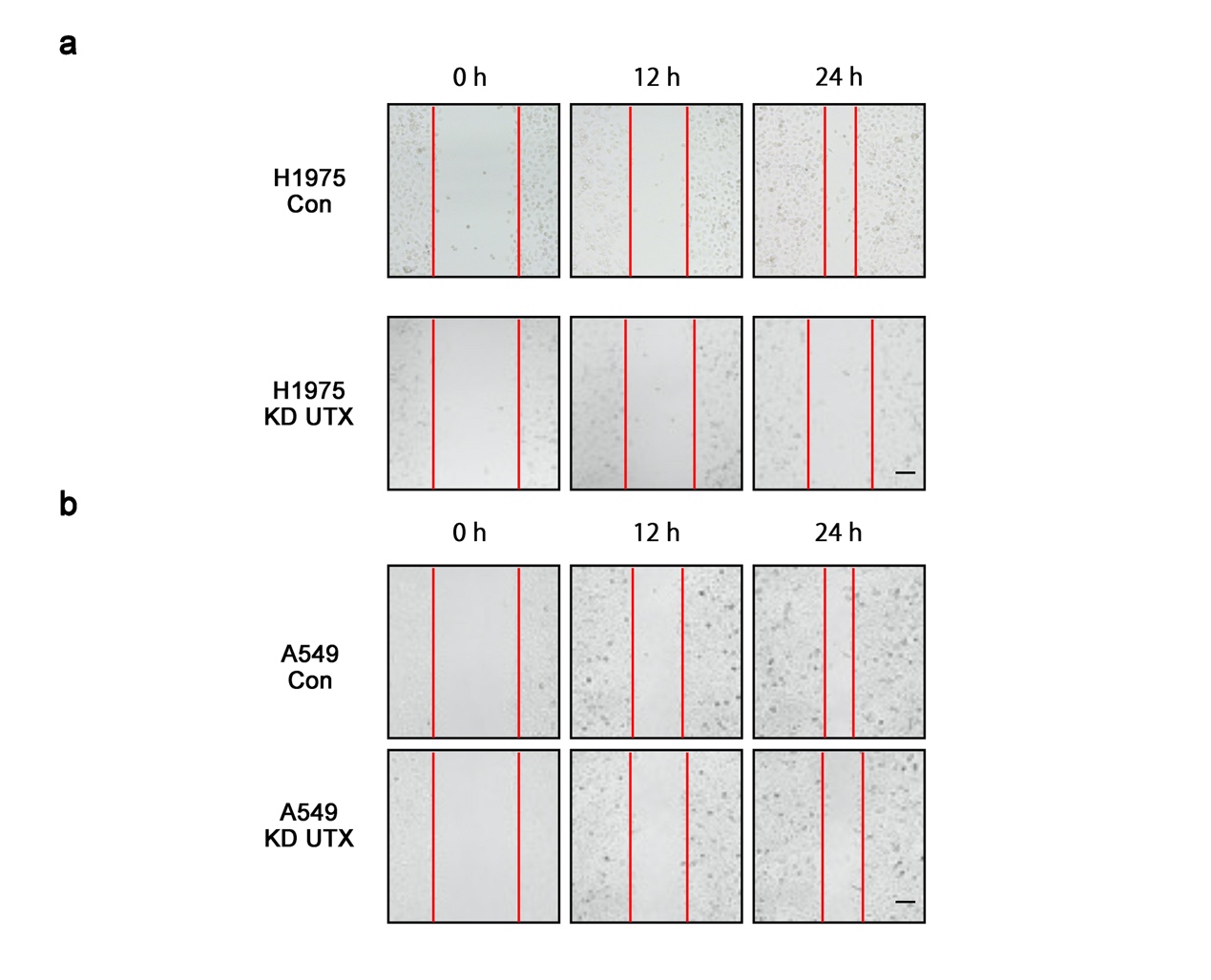
**

**Figure S1.** Representative results of wound healing assay. A, UTX knockdown suppressed migration of H1975. B, UTX knockdown suppressed migration of A549. Scale Bar, 100 μm.


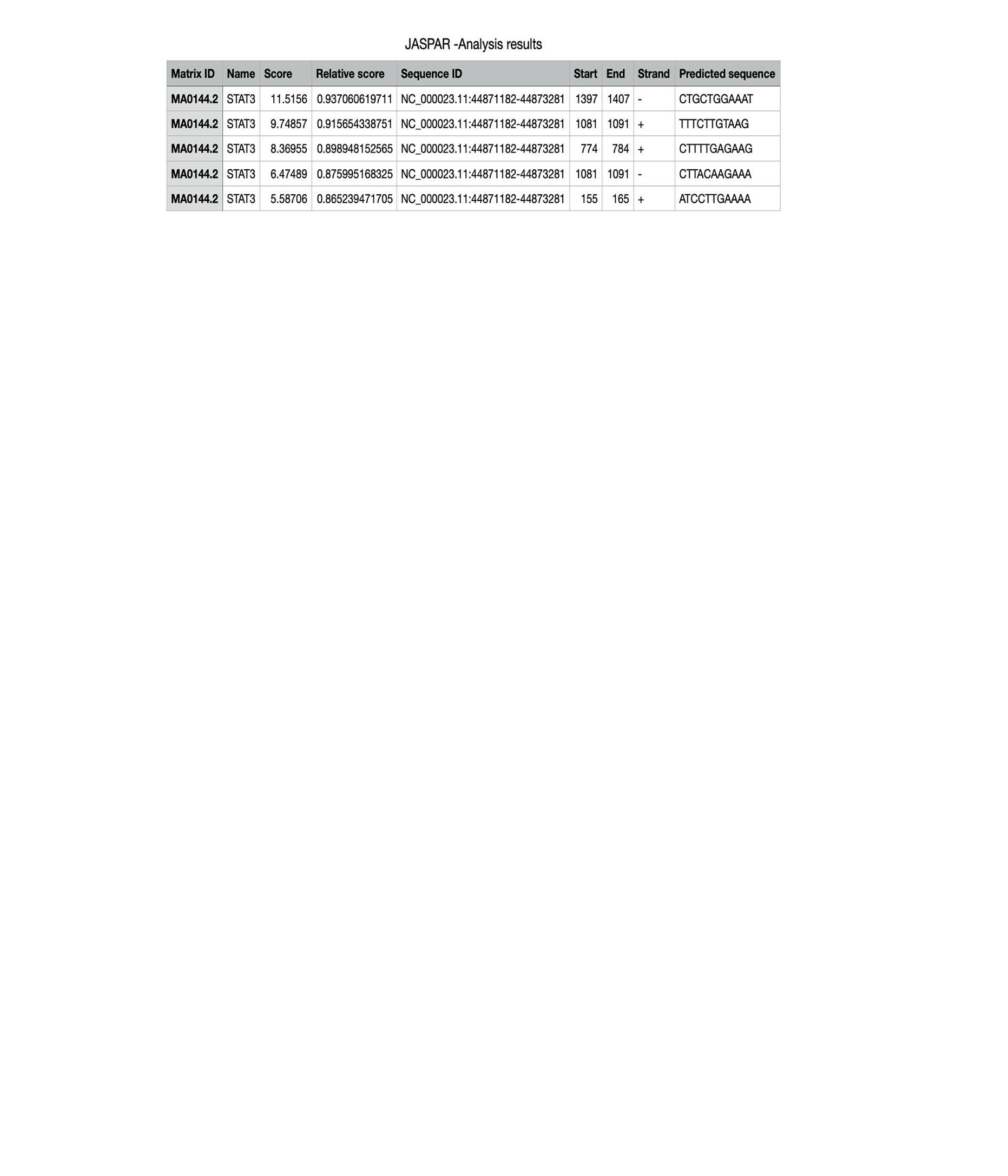


**Figure S2.** Five highest-rated predicted STAT3-binding sites on UTX promoter, as analyzed by JASPAR (http://jaspar.genereg.net/).

**Table S1. List of antibodies in the present study.**

| **Antigen** | **Cat. No** | **Company** | **Dilution** | **RRID** |
| --- | --- | --- | --- | --- |
| **Antibodies for western blot** | | | |  |
| UTX | A302-374A | Bethyl Laboratories | 1:1000 | AB_1907257 |
| UTX | 33510S | Cell Signaling Technology | 1:1000 | AB_2721244 |
| β-actin | A5316 | [Sigma-Aldrich](https://www.baidu.com/link?url=jLBCrnXLR4AqpW3VTXqYh5z2VA04R3qCzsqd5e2WA_CBDACu4pn_DV8fuP3jwxjT&wd=&eqid=fd4343d700013eb50000000359cf07ac) | 1:10000 | AB_476743 |
| p-EGFR | 3777S | Cell Signaling Technology | 1:1000 | AB_2096270 |
| EGFR | 2232S | Cell Signaling Technology | 1:1000 | AB_331707 |
| p-STAT3 | 9131S | Cell Signaling Technology | 1:1000 | AB_331586 |
| STAT3 | 4904S | Cell Signaling Technology | 1:1000 | AB_331269 |
| p-ERK | 4370S | Cell Signaling Technology | 1:1000 | AB_2315112 |
| ERK | 4695S | Cell Signaling Technology | 1:1000 | AB_390779 |
| p-AKT | 4058S | Cell Signaling Technology | 1:1000 | AB_331168 |
| AKT | 9272S | Cell Signaling Technology | 1:1000 | AB_329827 |
| H3 | 4499S | Cell Signaling Technology | 1:10000 | AB_10544537 |
| H3K27me3 | PTM622 | PTM Biolabs | 1:1000 | AB_2752230 |
| **Antibodies for immunohistochemistry** | | | |  |
| UTX | A302-374A | Bethyl Laboratories | 1:1000 | AB_1907257 |

**Table S2. Features and IHC score of paired NSCLC and paracancerous samples.**

| NO. | Sex | Age | EGFR status | Pathology | UTX IHC Score | |
| --- | --- | --- | --- | --- | --- | --- |
|  |  |  |  |  | Tumor | Para |
| 1 | F | 51 | 19-Del | ADC | 1.3 | 0.0 |
| 2 | M | 62 | WT | ADC | 1.0 | 3.2 |
| 3 | M | 63 | WT | ADC | 3.3 | 0.0 |
| 4 | M | 63 | WT | SCC | 2.7 | 3.7 |
| 5 | M | 60 | WT | ADC | 0.7 | 4.3 |
| 6 | F | 67 | NA | ADC | 2.7 | 3.9 |
| 7 | M | 63 | NA | ADC | 0.0 | 4.3 |
| 8 | M | 49 | WT | ADC | 0.0 | 0.0 |
| 9 | M | 62 | 19-Del | ADC | 0.0 | 3.7 |
| 10 | M | 64 | WT | SCC | 1.3 | 3.3 |
| 11 | M | 66 | WT | SCC | 0.0 | 2.7 |
| 12 | M | 52 | WT | SCC | 0.0 | 2.7 |
| 13 | M | 36 | WT | SCC | 1.3 | 4.3 |
| 14 | M | 58 | WT | SCC | 0.0 | 3.7 |
| 15 | M | 58 | WT | ADC | 0.0 | 4.0 |
| 16 | F | 50 | 19-Del | ADC | 1.0 | 5.1 |
| 17 | M | 62 | NA | SCC | 4.7 | 5.3 |
| 18 | M | 63 | WT | ADC | 2.0 | 5.3 |
| 19 | M | 62 | WT | SCC | 3.0 | 5.3 |
| 20 | M | 53 | NA | AD-SCC | 2.3 | 2.3 |
| 21 | M | 72 | WT | LCC | 0.0 | 4.7 |
| 22 | F | 69 | 19-Del/L858R | ADC | 0.7 | 5.0 |
| 23 | M | 57 | 19-Del | ADC | 3.7 | 4.3 |
| 24 | M | 58 | WT | SCC | 3.7 | 0.0 |
| 25 | M | 76 | WT | ADC | 3.7 | 3.3 |
| 26 | M | 55 | WT | SCC | 3.3 | 3.7 |
| 27 | M | 68 | WT | LCC | 3.0 | 4.3 |
| 28 | M | 61 | WT | SCC | 3.3 | 4.7 |
| 29 | M | 61 | WT | SCC | 4.0 | 5.0 |
| 30 | F | 62 | L858R | ADC | 3.3 | 4.0 |

F, female; M, male; WT, wild type; NA, not available; ADC, adenocarcinoma; SCC, squamous cell carcinoma; AD-SCC, adeno-squamous cell carcinoma; LCC, large cell carcinoma.

**Table S3. Features of 40 paired NSCLC and paracancerous samples.**

| NO. | Sex | Age | EGFR status | Pathology |
| --- | --- | --- | --- | --- |
| 1 | M | 57 | 19Del | ADC |
| 2 | M | 53 | WT | SCC |
| 3 | M | 58 | 19Del | ADC |
| 4 | F | 62 | 20Ins | ADC |
| 5 | M | 58 | WT | ADC |
| 6 | F | 51 | 19Del | ADC |
| 7 | M | 62 | WT | ADC |
| 8 | M | 63 | WT | SCC |
| 9 | M | 60 | WT | ADC |
| 10 | F | 67 | NA | ADC |
| 11 | M | 63 | NA | ADC |
| 12 | M | 49 | WT | ADC |
| 13 | M | 62 | 19Del | ADC |
| 14 | M | 64 | WT | SCC |
| 15 | M | 66 | WT | SCC |
| 16 | M | 52 | WT | SCC |
| 17 | M | 36 | WT | SCC |
| 18 | M | 58 | WT | SCC |
| 19 | F | 50 | 19Del | ADC |
| 20 | M | 62 | NA | SCC |
| 21 | M | 63 | WT | ADC |
| 22 | M | 62 | WT | SCC |
| 23 | M | 53 | NA | AD-SCC |
| 24 | M | 72 | WT | LCC |
| 25 | F | 69 | 19Del/L858R | ADC |
| 26 | M | 57 | 19Del | ADC |
| 27 | M | 58 | WT | SCC |
| 28 | M | 76 | WT | ADC |
| 29 | M | 55 | WT | SCC |
| 30 | M | 68 | WT | LCC |
| 31 | M | 61 | WT | SCC |
| 32 | M | 61 | WT | SCC |
| 33 | F | 62 | L858R | ADC |
| 34 | M | 65 | WT | SCC |
| 35 | M | 66 | NA | ADC |
| 36 | M | 64 | WT | ADC |
| 37 | M | 66 | WT | ADC |
| 38 | F | 52 | WT | ADC |
| 39 | M | 63 | WT | SCC |
| 40 | F | 37 | WT | ADC |

F, female; M, male; WT, wild type; NA, not available; ADC, adenocarcinoma; SCC, squamous cell carcinoma; AD-SCC, adeno-squamous cell carcinoma; LCC, large cell carcinoma.
